# Neurogenic regulation of group 2 innate lymphoid cells in asthma

**DOI:** 10.3389/fimmu.2026.1927691

**Published:** 2026-10-05

**Authors:** Takahiro Matsuyama, Nahal Emami Fard, Akimichi Nagashima, Roma Sehmi

**Affiliations:** 1 Respiratory Research Group, Department of Medicine, McMaster University, Hamilton, ON, Canada; 2 Department of Pulmonary Medicine, Graduate School of Medical and Dental Sciences, Kagoshima University, Kagoshima, Japan; 3 Firestone Institute for Respiratory Health, St. Joseph’s Healthcare, Hamilton, ON, Canada

**Keywords:** airway innervation, asthma, group 2 innate lymphoid cell, neuroimmune interactions, neuropeptide, neurotransmitter

## Abstract

Asthma is a chronic airways disorder characterized by reversible airflow limitation that is variable, airway hyperresponsiveness and bronchial inflammation and is associated with symptoms of wheeze, cough and productive mucus. Asthma can be phenotyped based on airway inflammation, where eosinophilic asthma is the most prevalent phenotype. Group 2 innate lymphoid cells (ILC2) play a central role in the pathogenesis of eosinophilic asthma by producing copious quantities of type 2 cytokines, such as IL-5 and IL-13, in an antigen-independent manner. ILC2 have been shown to promote airway inflammatory response in response to epithelial-derived cytokines (IL-25, IL-33, thymic stromal lymphopoietin and tumor necrosis factor-like peptide 1A (TL1A)), lipid mediators and hormones. Among soluble mediators, there is a growing body of data from murine and human studies showing that neurotransmitters and neuropeptides secreted by local nerve fibers contribute to the modulation of lung ILC2 activation in the pathogenesis of asthma. In this review, evidence supporting the relationship between ILC2 and neurogenic inflammation in asthma pathogenesis is discussed.

## 1 Introduction

Asthma is a heterogeneous airway disease characterized by airway inflammation and reversible airflow obstruction, causing symptoms such as cough, dyspnea and wheezing (1). Asthma is estimated to affect 334 million people globally (2). Currently, the number of patients continues to increase in many low- and middle-income countries (3). Asthma has a variety of inflammatory phenotype; eosinophilic, neutrophilic, mixed granulocytic, and pauci-granulocytic asthma (4). Among them, eosinophilic asthma is the most prevalent phenotype. Eosinophilia in asthma occurs as a result of allergic and non-allergic mechanisms, mainly associated with type 2 T helper (Th2) cells or group 2 innate lymphoid cells (ILC2), respectively (5).

ILC2 are lineage-negative cells that lack the rearranged antigen receptors expressed on adaptive immune B and T cells, and they are potent producers of IL-5 and IL-13 and can also produce IL-9 and, under specific stimulatory conditions, IL-4 (6, 7). Studies in humans and mice increasingly show that ILC2 functions are regulated by a variety of soluble factors including pro-inflammatory cytokines, lipid mediators, neuropeptides and sex hormones that contribute to the development of inflammatory responses (8, 9). Classically, neurogenic inflammation refers to inflammatory responses triggered by activation of sensory nerves and the subsequent release of neuropeptides, particularly tachykinins and calcitonin gene-related peptide (CGRP), resulting in vasodilation, plasma extravasation, edema, and immune cell recruitment. In addition, neurogenic inflammation can develop as a result of the activation of nerves by inflammatory mediators and irritants, such as prostaglandins, certain cytokines (IL-1β, TNF-α) and cigarette smoke (10). As such, neurogenic inflammation is implicated in the pathophysiology of inflammatory diseases, including asthma (11). Recently, increasing evidence has demonstrated interaction between ILC2 and neurons in various diseases. Neurons communicate with immune cells through the release of neurotransmitters and neuropeptides, which can modulate immune responses by influencing cytokine production and cellular activation. The focus of this review is the broader neuroimmune regulation mediated by neuron-derived signaling molecules. We will discuss the roles of neurotransmitters and neuropeptides in regulating ILC2 responses and effects on airway inflammatory responses in asthma.

## 2 Role of ILC2 in asthma pathophysiology

Airway inflammation is an important driver of asthma, characterized by the activation and infiltration of an array of innate and adaptive immune cells including macrophages, dendritic cells (DCs), mast cells, T lymphocytes, eosinophils, neutrophils, and basophils and ILC2 (12). Airway eosinophilic inflammation can arise by two different pathways, contributing to allergic and non-allergic asthma. Allergic asthma involves clonal expansion of Th2 cells stimulated by antigen-presenting including DC. This results in antigen-specific Th2 cells secreting IL-4, IL-5, IL-9 and IL-13 which promote eosinophil activation and recruitment into the airways, mucus hypersecretion, and synthesis of antigen-specific immunoglobulin E (IgE) by B cells. Moreover, synthesized IgE coats mast cells and basophils where subsequent cross-linking in response to antigen re-exposure triggers degranulation and release of histamine, leukotrienes and prostaglandins. This leads to the early and rapid phase of the allergic response, which is characterized by bronchoconstriction, enhanced vascular permeability and an increased pro-inflammatory cell recruitment in the lungs (12). Furthermore, lung ILC2 can interact with T cells through OX40L/OX40 signaling and produce IL-4, subsequently contributing to promote T cell-mediated acquired immunity (13). In addition, ILC2 are also involved in non-allergic eosinophilic inflammation in asthma. Air pollution, cigarette smoke, viruses and allergen proteases stress ciliated airway epithelial cells or activate them through pattern recognition receptors to release epithelial-derived alarmin cytokines, such as IL-25, IL-33, thymic stromal lymphopoietin (TSLP) and tumor necrosis factor-like peptide 1A (TL1A), which stimulate ILC2, subsequently producing large amounts of T2 cytokines that promote airway eosinophilia and mucus hypersecretion (14). In general, these environmental factors are known as triggers of asthma exacerbations (3, 9, 14–16). A growing body of evidence demonstrates that ILC2 are increased both in number and activation status in patients with asthma, particularly within the airways. In patients with eosinophilic and allergic asthma, circulating ILC2 frequencies and their capacity to produce T2 cytokines are elevated compared with healthy controls, and these increases are more pronounced in sputum and bronchoalveolar lavage fluid (BALF) (17–20). Higher ILC2 levels are associated with disease severity and key clinical biomarkers, including eosinophilia and fractional exhaled nitric oxide (FeNO), while showing an inverse correlation with lung function (20). Importantly, in severe asthma, ILC2 exhibit reduced sensitivity to corticosteroids. Experimental and clinical observations suggest that this steroid insensitivity is driven by the airway microenvironmental cues, where elevated levels of alarmins and related cytokines play a critical role. Combinations of IL-33, TSLP, and TL1A promote persistent ILC2 activation and sustain type 2 cytokine production despite corticosteroid treatment (21–23). These findings indicate that local cytokine networks within the airways not only amplify ILC2 responses but also contribute to steroid-resistant inflammation in severe eosinophilic asthma.

## 3 Neurogenic inflammation in asthma pathophysiology

In general, neuropeptides and neurotransmitters are released from central neurons and peripheral nerve fibers (24). Peripheral nerves are broadly classified into sensory and motor nerves, and the motor nerve system itself has two branches, autonomic and somatic nerves. The autonomic nervous system is further divided into sympathetic and parasympathetic branches (7). Airway innervation is mainly associated with sensory and parasympathetic nerves. It plays essential roles in defense mechanisms against environmental stimuli, viral and bacterial infections as well as regulation of breathing. Most of these nerve fibers travel to the airways through the vagal nerve (25). Sensory nerves are afferent, and most of the cell bodies are located in the jugular ganglia and nodose ganglia, and a few parts of them originate from the dorsal root ganglia in Th1-6 (25, 26). On the other hand, in parasympathetic nerves, the preganglionic parasympathetic nerves are localized in the dorsal motor nucleus of the vagal nerve and synapsed with cholinergic postganglionic neurons within the trachea wall and intrapulmonary airways. Postganglionic nerves have two subtypes, cholinergic and non-cholinergic nerves. Cholinergic postganglionic nerves are derived from the trachea wall and intrapulmonary airways, while non-cholinergic postganglionic nerves are derived from the myenteric plexus of esophagus (25, 27). On the other hand, sympathetic nerves are less prominent in the airways, and their innervation of human airway smooth muscle has not been demonstrated. However, sympathetic nerves may control tracheobronchial blood vessels (28). Specifically in asthma pathogenesis, neurogenic inflammation mediated by tachykinins, such as substance P and neurokinin A, can play a crucial role in initiating airway smooth muscle contractions and mucus hypersecretion (29). Indeed, it has been observed that levels of substance P in BALF of asthmatics is higher than that of healthy subjects and is further increased by antigen provocation (30). Meanwhile, sensory neurons release neuropeptides, including CGRP and substance P. Cholinergic neurons release a neurotransmitter ACh when synapsing, and non-cholinergic neurons release vasoactive intestinal peptide (VIP), a neuropeptide (25). In contrast, sympathetic neurons can produce a class of neurotransmitters known as catecholamines (**Figure 1**).

**Figure 1:**
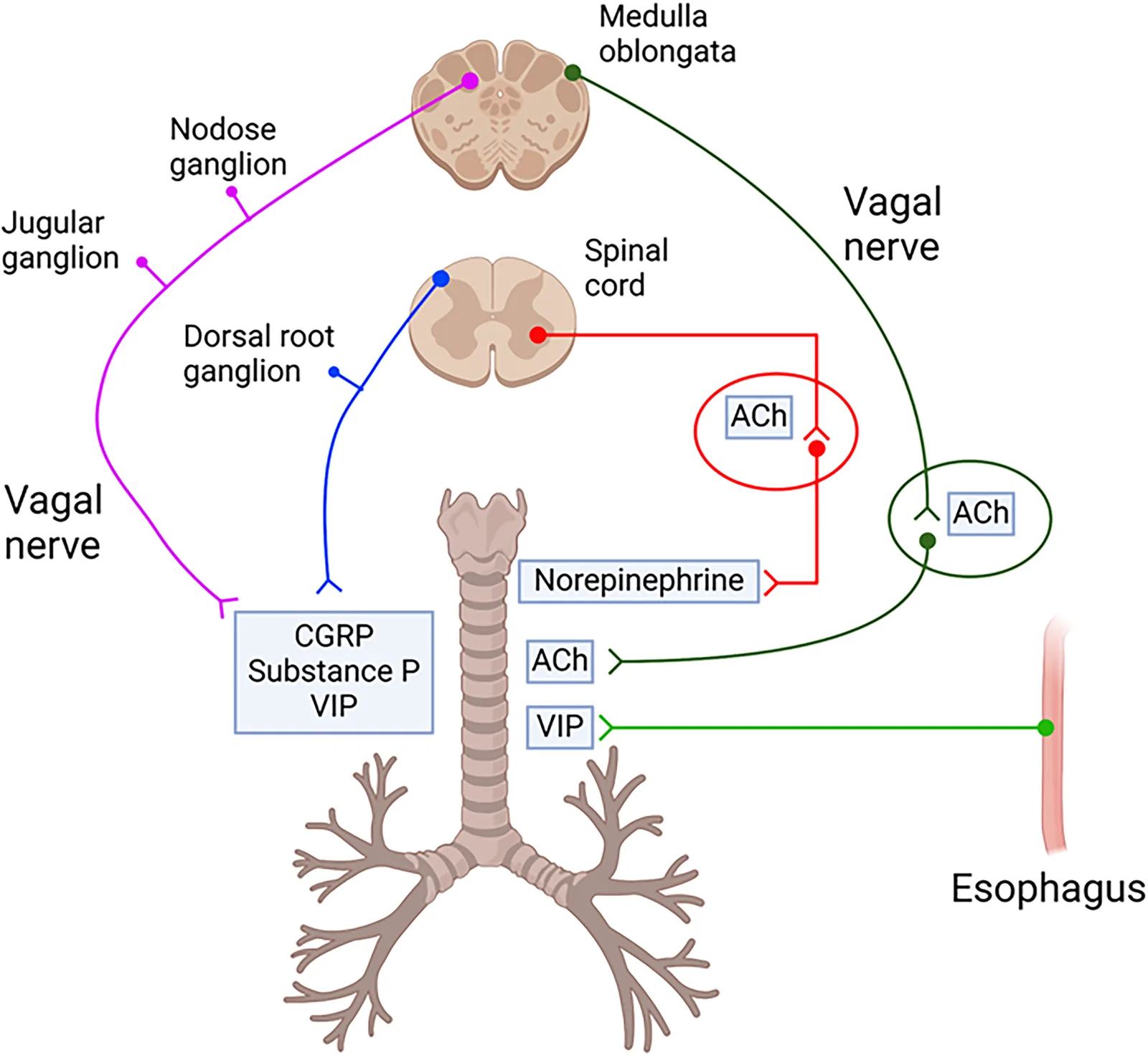
Airway innervation and released neuropeptide and neurotransmitter. Most of sensory and parasympathetic nerves travel to the airways through the vagal nerve derived from medulla oblongata. Also, a part of sensory nerve originates in the Th1–6 dorsal root ganglia. Sensory (blue and pink) and parasympathetic neurons (green) release neuropeptides including CGRP and Substance P, and ACh, neurotransmitter, respectively. A part of parasympathetic neurons (light green) resides in esophagus, releasing VIP. On the other hand, airway sympathetic neurons (red), which originate from the spinal cord, release norepinephrine, although they are less prominent. ACh, acetylcholine; CGRP, calcitonin-gene related peptide; VIP, Vasoactive intestinal peptide.

Airway sensory nerves are located close to airway eosinophils, and eosinophils contributed to altering sensory neuron structure, as increased airway eosinophilia positively correlated with nerve branching (31, 32). Indeed, patients with moderate asthma who exhibited persistent symptoms and elevated airway eosinophil counts showed a more pronounced increase in nerve length and branching compared to patients with mild intermittent asthma and healthy individuals (32). Also, eosinophils were recruited to the airways by allergen exposure, which increased release of substance P from sensory neurons, resulting in further bronchoconstriction (31). Therefore, these findings suggest that sensory nerves are activated by immune cells, including eosinophils, as well as irritants, leading to structural changes in the neurons and the release of neuropeptides such as substance P. On the other hand, administration of substance P directly acted on B cells isolated from naïve murine spleens to induce IgE production *in vitro*. In an *Alternaria*-induced model, sensory neuron-depleted mice exhibited a reduction in B cell numbers and airway hyperresponsiveness (AHR), while substance P treatment restored these parameters (33). Thus, sensory neuron-derived substance P can contribute to asthma pathogenesis via humoral immunity. These neuropeptides and neurotransmitters then go on to interact with immune cells, as well as structural cells including airway epithelial cells and smooth muscle cells (34). In this neuroimmune interaction, neuropeptides and neurotransmitters released from neurons modulate the functions of immune cells, whereas inflammatory mediators produced by immune cells affect neuronal activity, forming in a feedback loop (24). ILC2 are one of the immune cells that have been shown to be modulated by neuropeptides and neurotransmitters, as detailed below (8, 9).

## 4 Impacts of neuropeptides and neurotransmitters on ILC2 in asthma

Among immune cells interacting with neurons, ILC2 are primarily tissue-resident. In the lungs, which are highly innervated, immunostaining studies have demonstrated that ILC2 frequently co-localize with nerve fibers, with approximately 70% residing within 10 µm of nerve terminals (35). Together with the rapid responsiveness of ILC2 in mucosal tissues to changes in the local microenvironment, this anatomical proximity suggests that neuronal regulation of ILC2 activity may play an important role in inflammatory diseases, such as asthma. This section highlights human and animal studies focused on the effects of various neuropeptides and neurotransmitters on ILC2 in asthma pathophysiology. Here, Neuromedin-U (NMU), CGRP and VIP are classified as neuropeptides, while ACh, dopamine, norepinephrine, and serotonin are classified as neurotransmitters; each of these categories is further divided into three sections.

### 4.1 Activation of neuroimmune signals

#### 4.1.1 Neuromedin-U signaling

Neuromedin is a neuropeptide released from neurons, and classified into four groups; (a) Bombesin (Neuromedin-B (NMB and NMC), (b) Kassinin (NMK and NML), (c) Neurotensin (NMN) and (d) NMU and NMS (36). Among them, NMU is released from cholinergic and sensory neurons, and play an important role in diverse physiologic processes, including smooth muscle contraction, blood pressure control, food intake (satiety), stress response, intestinal motility, energy balance and immune responses. NMU receptors (NMUR) are divided into two subtypes; NMUR1 and NMUR2, expressed mainly in peripheral tissues and CNS, respectively (37, 38).

As for the immune cells, murine ILC2 have been shown to selectively express NMUR1 (35, 39, 40). Intestinal and lung ILC2 colocalize with cholinergic neurons that express NMU in murine tissues, suggesting that neurons have the capacity to directly act on ILC2 to provoke inflammation through NMU/NMUR1 signaling (39, 40). In an *in vitro* study using intestine and lung ILC2, NMU treatment induced a rapid and potent T2 cytokine and amphiregulin production and ILC2 proliferation (39, 41). On the other hand, ILC2 derived from NMU-treated *NMUR1*-knock out (KO) mice did not exert increased production of T2 cytokine and amphiregulin, compared with WT mice (39–41). In line with these findings, *in vivo* NMU intraperitoneal treatment induced in prompt production of T2 cytokine through the Ca^2+^-calcineurin-Nuclear factor of activated T-cells (NFAT) cascade and extracellular signal-regulated kinase (ERK)1/2 signaling pathway (39). Further, in a parasite-infected murine model, NMU intraperitoneal treatment resulted in provoking airway eosinophilia, as well as promoted worm expulsion (40). Interestingly, a recent study demonstrated that respiratory syncytial virus (RSV)-infected mice exerted increased production of NMU in the lungs and upregulated NMUR1 expression on ILC2. Additionally, the treatment with NMU exacerbated RSV-induced ILC2-mediated airway eosinophilic inflammation through Phosphatidylinositol 3-kinase (PI3K), mitogen-activated protein kinase (MAPK), and NFAT signaling (42). Collectively, NMU may have the critical effects on the development of viral-induced asthma exacerbation, as well as asthma pathophysiology.

Findings from a murine intestinal ILC2 study suggest that NMU may also exert immunoregulatory effects through induction of IL-10-producing ILC2, raising the possibility of a suppressive role in airway eosinophilic inflammation (43). In murine asthma models, a subset of lung ILC2 that produces IL-10 and highly expresses transcription factor Id3 but not FoxP3 has been identified (44). These IL-10^+^ ILC2 are called regulatory ILC2 (ILCreg) or ILC2_10_, attenuating airway inflammation (44–46). Recently, studies with ILC2reg and ILC2_10_ indicate that IL-10 production from ILC2_10_ stimulated by IL-4 requires additional transcription factors, Blimp-1 and c-Maf (46). However, whether NMU stimulation enhances IL-10 production in the human lung ILC2 is currently unknown, and further investigations are required.

Based on the above findings in mice, there are a growing number of reports on the effects of NMU in asthma in humans. In human blood, NMUR1 expression is observed on Th2 cell, type 2 cytotoxic T (Tc2) cell, ILC2 and eosinophils, whereas NMUR2 was undetectable in these cells (47). Also, no significant difference in NMUR1 expression in blood was detected between healthy subjects, patients with non-eosinophilic and eosinophilic asthma. Specifically, the NMUR1 expression on lung-derived Th2 cell and Tc2 cells were higher than those in blood-derived samples, where expression on lung-derived ILC2 was comparable to those in blood. In *in vitro* experiments, NMU treatment induces cell migrations and production of IL-5 and IL-13 in Th2 cell, Tc2 cell and ILC2 in dose-dependent manners, as well as mouse ILC2 data (47). Conversely, the additional treatment of PGD_2_ to NMU for 4 h exhibited a synergistic increase in IL-5 and IL-13 production in Th2 cell and ILC2. Additionally, significant stimulatory effects of alarmins in addition to NMU on IL-5 and IL-13 production by ILC2 were observed under stimulation with IL-25/IL-33/TSLP in Th2 cells and Tc2 cells, as well as under stimulation with IL-25/IL-33 and NMU in ILC2 for 48 h (47). These findings indicate that NMU has the pro-inflammatory effects in human subjects, contributing to pathogenesis of T2 airway inflammatory diseases including asthma and allergic rhinitis (**Figure 2A**).

**Figure 2:**
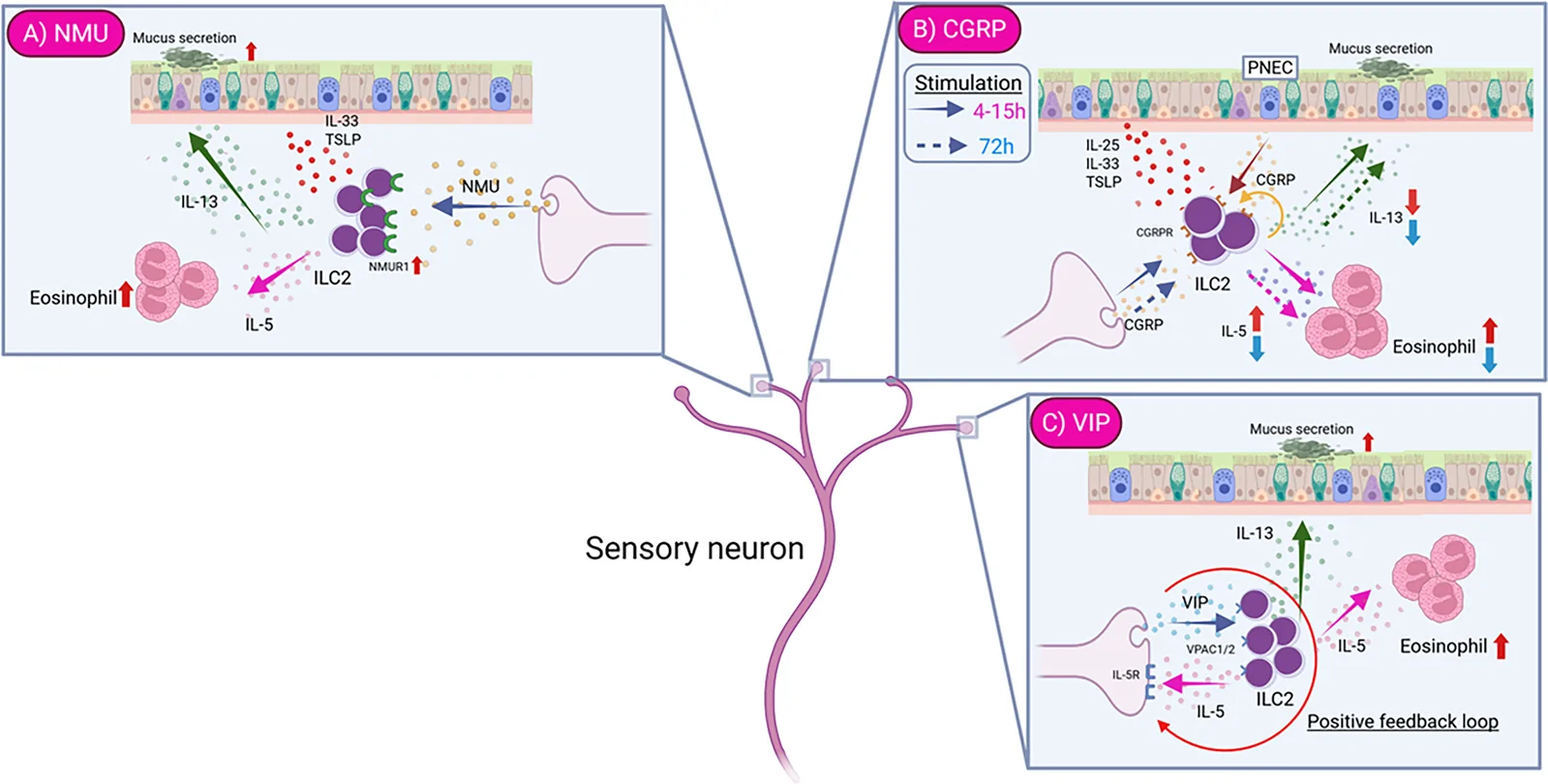
Perspectives on the impact of neuropeptides in ILC2-mediated eosinophilic airway inflammation. NMU **(A)**, CGRP **(B)**, and VIP **(C)** are shown as sensory neuron-derived neuropeptides. Epithelial-derived alarmins (IL-25, IL-33, TSLP) (red dots), PNEC-derived CGRP (brown), ILC2-derived CGRP (yellow) and cytokines (IL-5; pink and IL-13; green) are indicated separately. CGRP, calcitonin-gene related peptide; CGRPR, calcitonin-gene related peptide receptor; ILC2, group 2 innate lymphoid cell; NMU, Neuromedin-U; NMUR1, Neuromedin-U receptor 1; PNEC, pulmonary neuroendocrine cells; TSLP, thymic stromal lymphopoietin; VIP, vasoactive intestinal peptide; VPAC1/2, vasoactive intestinal peptide receptor type 1/2.

Recently, our group reported the influence of NMU in mild asthmatics. NMUR1 expression on sputum ILC2 was upregulated at 7 h post-allergen challenge, along with an increase in the numbers of total and IL-5/IL-13-producing ILC2. In addition, NMUR1-expressing ILC2 numbers correlated with sputum eosinophilia at 24 h post-allergen challenge. In contrast, NMU levels in sputum were not increased by allergen challenge. In an *in vitro* experiment using human blood ILC2, IL-5 and IL-13 production were enhanced by stimulation within 6 h with NMU and only detected at 24 h or later with IL-33 (48). The pro-inflammatory effects of NMU were found to be dependent on mainly MEK signaling, as well as NFAT and PI3K. These findings were also observed in a study in patients with allergic rhinitis (49). Patients with allergic rhinitis exhibited increased production of IL-5 and IL-13 from blood ILC2 stimulated with NMU, compared to healthy subjects, and NMU-induced activation of ILC2 was especially dependent on ERK signaling (49). On the other hand, dexamethasone treatment significantly attenuated production of IL-5 and IL-13 by ILC2 stimulated with NMU, *in vitro*. Interestingly, moderate/severe asthmatics exhibited lower expression of NMUR1 on sputum ILC2 and lower levels of NMU in sputum compared with mild asthmatics. Moreover, NMUR1^+^ ILC2 numbers had an inverse correlation with inhaled corticosteroid dose in the sputum of moderate/severe asthmatics (48). In contrast, the anti-inflammatory actions of glucocorticoids are associated with glucocorticoid receptor (GR)-mediated inhibition of MAPK (50). Of note, moderate/severe asthmatics use high-dose inhaled corticosteroids. In view of NMU activation of ILC2 via MEK upstream of MAPK, NMUR1 expression on sputum ILC2 and sputum NMU levels in moderate/severe asthmatics may be reduced by treatment with corticosteroids. Therefore, factors other than NMU may be involved in the pathogenesis of severe asthma requiring high-dose corticosteroids. In contrast, NMU-mediated stimulation may be more important in acute activation of ILC2 in allergic inflammatory responses in milder disease.

### 4.2 Suppressive neuroimmune signals

#### 4.2.1 Norepinephrine signaling

Norepinephrine is a neurotransmitter released primarily from the terminals of postganglionic sympathetic nerve fibers in peripheral nervous system (51). Receptors for norepinephrine have two isoforms including α and β adrenergic receptors (α_1_/α_2_ and β_1_/β_2_/β_3_). In the respiratory tract, inhaled β_2_ agonists are frequently used as a bronchodilator for asthma and COPD. Moreover, intramuscular epinephrine is administered as rescue medication for lethal asthma associated with anaphylaxis and angioedema, not routinely used for other asthma exacerbations (1).

Regarding effects of β_2_ adrenergic agonists on airway inflammation, in mice challenged with house dust mite (HDM), treatment with formoterol, a β_2_ adrenergic agonist, has been shown to suppress airway inflammation without reducing IL-5 and IL-13. Formoterol inhibited production of IL-6 and IL-23 and enhanced production of IL-10 from DCs stimulated with HDM *in vitro* (52). Also, in an Ovalbumin (OVA)-challenged mouse model, oral administration of procaterol, another β_2_ adrenergic agonist, resulted in a decrease in the number of eosinophils in BALF without affecting IL-13 and AHR (53). Thus, the murine studies demonstrated that the anti-inflammatory effects of β_2_ adrenergic agonist were mediated through cytokines from DCs. Previous allergen-challenge studies in patients with asthma have reported inconsistent effects of salmeterol on eosinophilic inflammation: some demonstrated suppression of post-challenge eosinophil increases in BALF and sputum, whereas others showed no significant effect (54). Consistently, an early observational study also found that salmeterol treatment did not reduce eosinophil numbers in BALF or sputum (54). Taken together, these findings from murine and asthma patients suggest that although β_2_-adrenergic agonists are highly effective bronchodilators, their anti-inflammatory effects are limited. Furthermore, the FDA currently contraindicates LABA monotherapy without inhaled corticosteroids because of an increased risk of asthma-related death (55). Accordingly, the GINA guidelines recommend inhaled corticosteroids either alone or in combination with LABAs for asthma management (1).

*ADRB2* encodes a β_2_ adrenergic receptor. *ADRB2* was highly expressed on ILC2 derived from murine lungs and intestines. Also, human lung ILC2 exhibited a higher expression of *ADRB2* compared with blood ILC2 or CD4^+^ T cells. In the intestines, ILC2 are colocalized with adrenergic nerves, suggesting that they may respond to neuron-derived norepinephrine. A similar mechanism may also operate in the lungs. In a comparative study using intranasal IL-33-treated *Adrb2*-KO and WT mice, *Adrb2*-KO mice exhibited increased frequencies of lung ILC2 compared with WT mice. Furthermore, in an IL-33-administered model, treatment with salmeterol reduced ILC2 proportion and intracellular IL-5 and IL-13 production by ILC2 both in lungs and intestines (56). These murine findings suggest that β_2_-adrenergic receptor signaling in ILC2 contributes to the negative regulation of eosinophilic inflammation (**Figure 3A**). However, as noted above, in human studies, adrenaline has been shown to contribute to bronchodilation in asthma, while there is no evidence that directly suppress airway inflammation. Unlike murine models with specific experimental interventions, humans are exposed to a wide variety of environmental factors, which may confound the potential inhibitory effects of adrenaline on airway inflammation. On the other hand, a previous parallel double-blind study in patients with mild asthma showed that an initially observed improvement in AHR with salmeterol was attenuated following repeated treatment. This indicates that prolonged or excessive use of β_2_-adrenergic receptor agonists, including LABA and short-acting β2 agonists (SABA), leads to desensitization of β_2_ adrenergic receptors and subsequent tolerance to their bronchodilatory effect (57). Therefore, it may be difficult to evaluate airway inflammation by using airway ILC2 from asthmatics under treatment with LABA or SABA alone, given ethical issues. To evaluate the anti-inflammatory effects of β_2_ adrenergic agonists on human ILC2, blood-derived ILC2 isolated from allergen-exposed, steroid-free patients with allergic asthma could be stimulated with β_2_-adrenergic agonists *in vitro*, followed by assessment of T2 cytokine production although the effect of this treatment on disease control is likely marginal.

**Figure 3:**
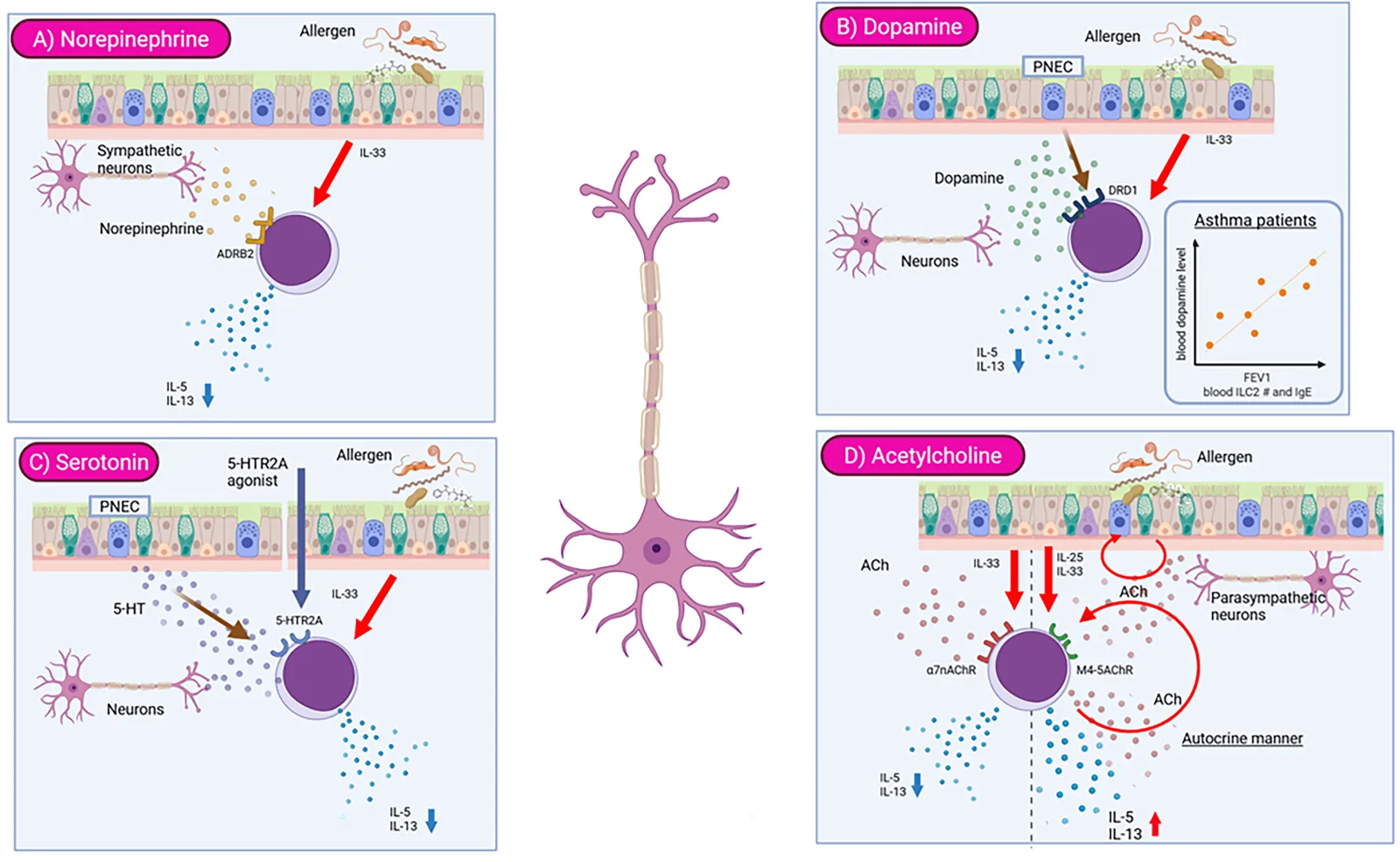
Perspectives on the impact of neurotransmitters in ILC2-mediated eosinophilic airway inflammation. Norepinephrine (yellow) **(A)** is derived from sympathetic neurons. Dopamine (green) **(B)** and Serotonin (purple) **(C)** are derived from neurons. Acetylcholine (orange) **(D)** is derived from parasympathetic neurons. Epithelial-derived alarmins (IL-33) (red), PNEC-derived neurotransmitters (brown) and ILC2-derived cytokines (blue) are indicated separately. ACh, acetylcholine; DRD1, Dopamine receptor D1; ILC2, group 2 innate lymphoid cell; 5-HT, 5-hydroxytryptamine; 5-HTR2A, 5-HT2A receptor; α7nAChR, α7 nicotinic acetylcholine receptor; M4-5AChR, muscarine 4–5 acetylcholine receptor.

#### 4.2.2 Dopamine signaling

Dopamine is a neurotransmitter that is synthesized in both the central nervous system and peripheral tissues including blood vessels, lungs, kidneys, heart, retina, and adrenals. Also, pulmonary neuroendocrine cells (PNECs) within the lungs may release dopamine. It plays an essential role in regulation of reward responses, movement, cognition and homeostasis of peripheral organs regulating blood pressure, renal activity, and intestinal motility (58–60). Dopamine deficiency is a known cause of Parkinson’s disease (PD). Dopamine receptors (DR) have five subtypes and are often grouped into stimulatory D1-like DR (D1 and D5) and inhibitory D2-like DR (D2, D3, and D4) (59). A variety of immune cells including DCs, T cells and ILC2 expresses all types of DR.

Murine and human ILC2 express DRD1 (dopamine receptor D1). Moreover, murine lung ILC2 exhibited higher expression of DRD1, compared with other immune cell types in the lung (61). With regards to the reported association between dopamine and airway inflammation, a study using OVA-induced T-cell receptor (TCR)-transgenic mice demonstrated that treatment with a DRD1 antagonist attenuated OVA-induced airway neutrophilic inflammation via reduction of IL-17-derived Th17 cells. In contrast, airway eosinophilic inflammation was not suppressed by treatment with DRD1 antagonist (62). Additionally, allergen-induced young mice, but not adult mice, exhibit Th2-mediated airway eosinophilic inflammation via dopamine/DRD4 pathway (63). On the other hand, Cao et al. reported that dopamine treatment suppressed this ILC2-driven airway eosinophilic inflammation in an IL-33-induced murine model. Furthermore, both systemic and ILC2-specific deletion of *Drd1* augmented ILC2 response and airway eosinophilic inflammation in this model (61). Moreover, A comparative study, involving patients with allergic asthma reported lower plasma dopamine levels compared with healthy controls. These studies also showed that plasma dopamine levels positively correlated with FEV_1_ and negatively correlated with blood ILC2 counts and plasma IgE levels (61). Interestingly, patients with asthma have also been shown to have an increased risk of developing PD later in life, with asthma severity appearing to positively correlate with the subsequent risk of PD development (64). Therefore, reduced dopamine levels may contribute to ILC2-driven airway inflammation, thereby promoting asthma pathophysiology as well as increasing susceptibility to PD (**Figure 3B**). However, direct evidence regarding the pathophysiology of asthma in humans remains limited, and further investigations are required.

#### 4.2.3 Serotonin signaling

Serotonin (5-hydroxytryptamine: 5-HT) is a monoamine neurotransmitter which plays key roles in the regulation of essential body functions including regulation of mood and the reward system, perception, anger, aggression, appetite, memory, sexual desire and attention (65). Dysregulation of the serotonergic system has been implicated in the pathogenesis of many psychiatric and neurological disorders, and selective serotonin reuptake inhibitors (SSRIs) as well as serotonin–norepinephrine reuptake inhibitors (SNRIs) are widely used in clinical practice for the treatment of depression and anxiety disorders (66). Although 5-HT is known to act on the central nervous system, the majority of 5-HT (~95%) in the body is found in the periphery, especially gastrointestinal tract such as enterochromaffin cells (67). In addition, PNEC in the airway epithelium are also capable of secreting 5-HT (60). To date, 14 serotonin receptors classified into seven receptor subfamilies have been identified throughout the body and brain (65, 68). Among them, the 5-HT2A receptor (5-HTR2A) is expressed on immune cells, including CD4^+^ T cells, macrophages, and eosinophils, as well as structural cells such as airway epithelial and smooth muscle cells. Furthermore, a recent study demonstrated that 5-HTR2A is expressed on both murine and human ST2^+^ ILC2, with higher expression levels than those observed in other immune cell populations (69).

With regards to asthma pathophysiology, a study using an OVA-challenged murine model demonstrated that treatment with a 5-HTR2A agonist attenuated effects on airway eosinophilia and AHR (70). Similarly, in a papain (protease allergen)-induced model, 5-HT treatment attenuated ILC2-driven airway eosinophilic inflammation. Additionally, treatment with 5-HTR2A agonist reduced numbers of eosinophils and ILC2 in lungs and BALF in a papain-induced murine model, resulting in suppression of airway eosinophilic inflammation. Consistent with these findings, an *in vitro* study using IL-33-stimulated murine ILC2 demonstrated that treatment with a 5-HTR2A agonist suppressed IL-5 and IL-13 production without affecting cell viability (69). In contrast to mice treated with a 5-HTR2A agonist, papain-induced mice with ILC2-specific deletion of HTR2A did not exhibit attenuated airway eosinophilic inflammation (69). These findings from murine asthma models suggest that 5-HT plays an important role in the suppression of ILC2-mediated airway eosinophilic inflammation solely through 5-HTR2A signaling pathway. In contrast, platelet-derived 5-HT has been reported to enhance airway eosinophilic inflammation by increasing the capacity of DCs to prime Th2 cell in an OVA-challenged murine model (71). Collectively, these murine studies suggest that 5-HT exerts both suppressive and proinflammatory effects on different cells involved in eosinophilic inflammation, thereby contributing to asthma pathophysiology, and that the effects of 5-HT may vary depending on its source and target cells (**Figure 3C**).

In asthma patients, 5-HT levels in BALF were increased by allergen challenge, while the serum levels were decreased. These findings suggest that inhaled allergen challenge may induce 5-HT translocation from the circulation to the airways. Consistent with this, 5-HT levels in BALF were higher in stable asthmatics than healthy subjects (71). In contrast, higher levels of 5-HT in plasma are also found in symptomatic asthmatics compared with asymptomatic ones. Additionally, levels of plasma 5-HT had a negative correlation with FEV_1_ in symptomatic asthmatics and a positive correlation with asthma severity (72). Taken together, these human findings suggest that circulating 5-HT, likely derived from platelets, plays a significant role in asthma pathogenesis. However, current clinical evidence is limited to measurements of circulating or airway serotonin levels and does not allow precise determination of serotonin origin. Therefore, further studies are required to clarify the relative contributions of platelet- and neuron-derived serotonin to airway inflammation in human asthma.

### 4.3 Context-dependent signals

#### 4.3.1 Acetylcholine signaling

ACh is a primary neurotransmitter released from postganglionic parasympathetic neurons in the airways (73). ACh binds to two types of receptors, nicotinic receptors (nAChRs) and muscarinic receptors (mAChRs). In the context of asthma pathophysiology, ACh causes not only airway smooth muscle contraction and mucus secretion through mAChRs, but also airway inflammation and remodeling. There are five subtypes (M1-5) mAChRs, where M1–3 receptors are expressed on lung structural and inflammatory cells, including macrophages and neutrophils (73, 74). Specifically, M3 receptor (M3R) plays an important role in airway smooth muscle contraction, while M2R, which is located on postganglionic nerves, inhibits ACh release from postganglionic nerve terminals thereby acting as negative feedback to prevent overexuberant inflammation (74). However, in asthma patients and allergen-challenged animal models, M2R has been shown to be dysfunctional. Major basic protein (MBP) derived from human eosinophils has been shown to cause the dysfunction of M2R (75). Consequently, dysfunctional M2R cannot control ACh release from postganglionic neurons and results in further increased ACh release, contributing to aggravation of AHR and mucus hypersecretion (76). In clinical practice, long-acting muscarinic receptor antagonists (LAMA) are approved for asthma and COPD. Current available LAMAs show similar affinity to M1-3R but also show greater kinetic selectivity for M3R and M1R than M2R due to a longer half-life of dissociation (73). LAMA is believed to act as a bronchodilator and to exert an inhibitory effect on mucus secretion. In fact, in the study of uncontrolled asthmatics treated with inhaled corticosteroid (ICS), the addition of tiotropium, a LAMA, improved symptoms and lung function (77). Moreover, the study of asthmatics poorly controlled by ICS and long-acting β_2_ agonist (LABA), demonstrated that the addition of tiotropium resulted in a reduced risk of severe exacerbation (78).

In murine models, there are growing reports of inhibitory effects of LAMAs on airway eosinophilic inflammation (79, 80). Regarding ILC2-mediated airway eosinophilic inflammation, basophils express M3R and upon activation following a papain-challenge, basophil-derived IL-4 can activate ILC2-derived cytokine expression, subsequently leading to airway inflammation (81). In a papain-induced model, it was demonstrated that tiotropium suppresses ILC2-driven airway eosinophilic inflammation by regulating basophil-derived IL-4 via M3R on basophils (82). Additionally, a recent study demonstrated that *Alternaria* administration directly acts on airway epithelial cells to induce ACh and IL-33 release *in vitro* and *in vivo* and that these findings were suppressed by treatment with M3R antagonist including tiotropium, subsequently regulating ILC2-driven airway eosinophilic inflammation (83). Thus, LAMA might have an indirect inhibitory effect on ILC2-mediated airway eosinophilic inflammation by acting directly on various sites. The findings from murine models and experimental studies suggest that ACh may act on ILC2 both directly and indirectly via the mAChR, thereby promoting the induction of airway inflammation. In contrast, the mechanism of anti-inflammatory effects of LAMA in human asthma remains unknown.

nAChRs have seven α-like subunits (α1-7, α9, α10) and four β subunits (β1-4) in mammals, and they are expressed on various cells, such as airway epithelial cells and immune cells, contributing to putative anti-inflammatory effects (84). OVA-challenged mice experienced suppression in airway eosinophilic inflammation and AHR following treatment with nAChR agonists (85). On the other hand, in the study of blood basophils from patients with mild allergic asthma, nAChR on basophils was found to express α4, α7 and α1/α3/α5 subunits. The treatment with ASM-024, a synthetic nicotinic ligand, inhibited an enhanced CD203c expression, indicative of basophil activation, and histamine release by basophils stimulated with anti-IgE. The inhibitory effects of ASM-024 were restored by the treatment of muscarinic and nicotinic antagonists. Additionally, ASM-024 inhalation significantly inhibited CD203c expression on blood basophils collected 24 h after allergen challenge. These findings suggest that nicotine can attenuate basophil activation and histamine release, regulating allergic asthma symptoms (86). However, the effects of nicotinic agonist on asthma symptoms have not been reported to date.

As for the association between ILC2 and ACh receptor, ILC2 express α7nAChR. In an *in vitro* study using IL-33-treated lung ILC2 isolated from mice, treatment with an α7nAChR agonist suppressed T2 cytokine production (IL-5 and IL-13) and cell viability, while in an *in vivo* study using an IL-33 or *Alternaria*-induced murine model, treatment with an α7nAChR agonist suppressed ILC2-mediated airway eosinophilic inflammation and AHR (87, 88). Furthermore, OVA-challenged *Chrna7* (encoding α7nAChR)-KO mice exerted increased lung ILC2 numbers and worsened allergic inflammation, compared to wild type (WT) mice (89). In a humanized ILC2 murine model treated with IL-33, the α7nAChR agonist treatment exhibited a reduction in numbers of ILC2 and eosinophils in BALF and lessened the degree of AHR (87). Therefore, α7nAChR activation has been suggested to have an inhibitory effect on ILC2.

In a *N. brasiliensis*-infected murine model, treatment with ACh resulted in an increase in numbers of ILC2 and eosinophils and the production of T2 cytokines from lung ILC2 (90). It was noted that ILC2 elicited an elevated expression of choline acetyltransferase (ChAT), the enzyme that synthesizes ACh. In an *in vitro* study, ChAT expression in ILC2 was enhanced by exposure to IL-25 and IL-33, and was further augmented by the addition of IL-2. Mice that were deficient for ChAT specifically in ILC2 also exhibited delayed worm expulsion and less airway eosinophilic inflammation, including T2 cytokine production and lung ILC2 proliferation, following subcutaneous inoculation with *N. brasiliensis*, compared to WT mice (91). However, intranasal local administration with *Alternaria* did not affect airway eosinophilic inflammation, but enhanced airway neutrophilic inflammation, evidenced by the increase in neutrophil numbers in lung tissue and BALF, along with an increase in expression of the neutrophil chemoattractants CXCL1 and CXCL2. By contrast, in an *Alternaria*-induced mouse model, depletion of ACh by acetylcholinesterase (AChE), an enzyme that catalyzes the breakdown of acetylcholine, attenuated ILC2-mediated airway eosinophilia, whereas it enhanced airway neutrophilic inflammation (92). Intestinal IL-17RB^+^ ILC2 migrates to the lungs following early activation during helminth infection, where they upregulate ST2 expression, acquire a lung-resident ILC2 phenotype, and contribute to lung inflammation (93, 94). Some of intestinal-derived “ST2^+^ ILC2” not only return to the intestines but also migrates to other tissues via the circulation, eventually disseminating systematic inflammation (94, 95). Thus, based on data from murine models of asthma and helminth infection, cholinergic regulation of ILC2s may involve multiple cellular sources of ACh. Neurons are an established source of ACh in neuroimmune communication, whereas these murine findings suggest that ILC2-derived ACh may contribute to ILC2 proliferation and expansion through ChAT-ACh-AChR signaling, potentially through an autocrine mechanism. Furthermore, ILC2 derived from the intestine, rather than the lung, may migrate to the lungs to affect airway inflammation (94). Moreover, recently, feeding was shown to trigger ILC2-driven airway T2 response in mice treated with intranasally IL-33 or papain. Food intake increased neuronal activity in the dorsal motor nucleus of the vagus, which generated preganglionic parasympathetic signals, and elevated plasma acetylcholine levels. ACh subsequently stimulated ILC2 through M4R in this model (96). These findings from murine intestinal study suggest that a gut-brain-lung parasympathetic pathway induces ILC2-mediated airway inflammation. Whether this mechanism contributes to disease severity or postprandial worsening of symptoms in human asthma remains to be determined. This section indicated that ACh is released from postcholinergic nerve and ILC2. However, the differences also remained unclear. Therefore, there are still many challenges remaining regarding ACh.

Based on the findings described above, ACh appears to exert bidirectional effects on airway inflammation through distinct receptor subtypes (**Figure 3D**). Thus, the overall impact of ACh in asthma may depend on the balance between these receptor pathways. However, the relative affinity of ACh for each receptor subtype and their respective contributions to disease pathogenesis remain unclear and warrant further investigation.

#### 4.3.2 Calcitonin-gene related peptide signaling

CGRP is a neuropeptide released from sensory neurons as well as non-neuronal cells, such as endothelial cells, adipocytes, B cells and macrophages. It has a potent vasodilatory function and is involved in protective mechanisms that are important in physiological and pathological conditions especially related to the cardiovascular system and wound healing (97). In clinical practice, anti-CGRP receptor monoclonal antibody and anti-CGRP ligand monoclonal antibody have been approved for the prevention of episodic and chronic migraine by the United States Food and Drug Administration (FDA) (98). CGRP receptor consists of three subtypes: calcitonin-like receptors (CALCLRs) form a complex with two accessory proteins, receptor activity modulating protein (RAMP1) and CGRP-receptor component protein (RCP) (99).

Airway sensory neuron silenced by QX-314, a permanently charged quaternary derivative of lidocaine, exerted a reduction in capsaicin-induced CGRP release in OVA-challenged mice (100). Treatment with capsaicin, a transient receptor potential vanilloid 1 (TRPV1) agonist, significantly increased CGRP levels in BALF, which were further enhanced, along with IL-5 and eosinophilia, in response to OVA challenge (100). Furthermore, mice treated intratracheally with 2-chloroacetophenone, a transient receptor potential ankyrin 1 (TRPA1) agonist, exhibited potent elevated levels of CGRP in BALF, whereas 2-chloroacetophenone-induced CGRP release was significantly reduced in *Trpa1*-KO mice (101). Of note, dibutylphthalate, a synthetic plasticizer, was recently shown to significantly enhance the OVA-induced CGRP production from TRPV1^+^ neurons through Ca^2+^ influx in the lungs (102). TRPA1 and TRPV1 are members of TRP channel. In general, TRP channels are mainly localized in nerve endings of sensory neurons, playing roles in sensory perception and cellular physiology through activation by physical (temperature, voltage, pressure and tension) and chemical stimuli (103). Specifically, TRPV1 is found to act as a heat sensor and TRPA1 as a sensor of both heat and cold in humans (103, 104). Recent research demonstrated that cold air exposure exhibited an increase in CGRP mRNA expression in lungs of papain-treated mice, while CGRP mRNA expression was comparable between *Trpa1*-WT mice and KO mice. On the other hand, *Trpa1*-WT mice treated with papain exhibited a further increase in airway eosinophilic inflammation by cold air exposure, while *Trpa1*-KO mice failed to augment that (105). Generally, cold air exposure exacerbates asthma symptoms, and these findings indicate that CGRP and TRPA1 channel contribute to the pathogenesis of asthma exacerbation, respectively.

On the other hand, CGRP is also produced by PNEC as well as sensory neurons (106, 107). PNEC are the only innervated airway epithelial cells, accounting for less than 1% of the total lung epithelial cells and acting as airway sensors that trigger immune responses via neuropeptide release including CGRP (108). ASCL1 is a master regulator of neurogenesis in vertebrates and is essential for the development of PNEC (106, 109). Interestingly, in an OVA-challenged murine model, *Ascl1*-KO mice exhibited lower numbers of ILC2, Th2 cells and eosinophils, suppressing airway inflammation. In addition, in lungs of *Ascl1*-KO mice, expression of *Calca*, encoding CGRP, was significantly lower than in lungs of WT mice after OVA challenge. PNEC have been shown to reside in proximity to ILC2 in naïve mice (106). These murine findings demonstrate that PNEC are the source of CGRP in allergen-induced immune responses, and that PNEC have the potential to act on ILC2 to stimulate cytokine production through CGRP stimulation (106). In an OVA-challenged murine model, treatment with dibutylphthalate resulted in an increase in lung ILC2 frequencies, which were suppressed by olcegepant, a CGRP receptor antagonist (102). OVA-challenged mice treated with rimegepant, another CGRP receptor antagonist, also experienced a decrease in ILC2 numbers and their capacity to produce IL-5 (107). Therefore, blockade of CGRP to be shown to suppress ILC2 activation and ILC2-mediated airway inflammation.

Murine and human ILC2 express CGRP receptors, *Calcrl* and *Ramp1*, as was identified by qPCR and single-cell RNA-seq (106, 110). Additionally, an *in vitro* study demonstrated that CGRP treatment could trigger IL-5 production from murine lung ILC2 stimulated with IL-7/IL-33 ± IL-25, but not with IL-7 alone, for 6-15 h, whereas IL-13 production was reduced by 6 h-stimulation with CGRP/IL-7 regardless of the presence of IL-33 (106, 110). These *in vitro* findings are supported by another study showing that add-on treatment with CGRP resulted in an increased production of IL-5 and a decreased production of IL-13 in lung ILC2 pre-stimulated with IL-33 alone for 4 h (111). However, contrary to brief stimulation with CGRP, long-term, 3 day-stimulation with CGPR/IL-7 ± IL-33 decreased IL-5 production, as well as IL-13 production derived from murine lung ILC2 (110, 112). In mice treated with IL-33 for 3 days or *Rag1*-KO mice challenged with *Alternaria* for 4 days *in vivo*, CGRP treatment attenuated ILC2-mediated airway eosinophilic inflammation and AHR (110–112). One of the mechanisms involves β-CGRP (encoded by *Calcb*, distinct from α-CGRP encoded by *Calca*) produced by vagal sensory neurons, in which the Janus kinase 1 (JAK1) signaling promotes *Calcb* expression, leading to suppression of ILC2-driven airway eosinophilic inflammation (112). Taken together, the conflicting effects observed with CGRP may primarily reflect time-dependent regulation. Thus, current evidence suggests that CGRP functions as a pleiotropic mediator whose net effect depends on the inflammatory microenvironment rather than being uniformly pro- or anti-inflammatory.

Recently, phosphatase of activated cells 1 (PAC1), identified as negative regulators of MAPK, has been found to play an important role in CGRP signaling within ILC2 (113). In an *Alternaria*-induced model, *Pac1*-KO mice exhibited enhanced ILC2-driven airway eosinophilic inflammation, compared to WT mice. Furthermore, ILC2 with specific deletion of PAC1 (*Pac1*^-/-^ ILC2) elicited an augmented effect of CGRP on proliferation and secretion of IL-13 from ILC2 both *in vivo* and *in vitro*. Therefore, PAC1 might be involved in some mechanism by which CGRP suppresses IL-13 production from ILC2. Intriguingly, under CGRP stimulation, *Pac1*^+/+^ ILC2 experienced a greater increase in chromatin accessibility of *Calca* than *Pac1*^-/-^ ILC2 (113). This indicates that ILC2 may be capable of producing CGRP themselves, potentially forming an autocrine positive feedback loop. Consequently, these findings from murine studies suggest that CGRP has stimulatory and inhibitory effect on ILC2-derived IL-5 production at different time points and suppressive effect on ILC2-derived IL-13 production through PAC1 at all time points (4 h and 3 days) and that ILC2 themselves produce CGRP through PAC1 in addition to neurons (**Figure 2B**). However, CGRP derived from PNEC can be expected to directly act on airway ILC2, while the impact of sensory neurons or ILC2-derived CGRP on airway ILC2 has not been investigated.

In a human study, levels of *Pac1* expression in blood ILC2 were significantly reduced in grass-pollen allergic subjects, compared to non-allergic subjects. Also, the *Pac1* expression level in blood ILC2 negatively correlated with ILC2 numbers and IL-13 expression levels, while significantly positively correlated with *Calca* levels (113). Unfortunately, these data are for grass-pollen allergic patients, and no studies have been done thus far have looked at the role of CGRP in ILC2 function in the context of human asthma. Therefore, the translational relevance of the murine findings remains to be established.

#### 4.3.3 Vasoactive intestinal peptide signaling

VIP is an immunomodulatory neuropeptide which is distributed in the central and peripheral nervous system and is synthesized by immune cells which also express VIP receptors, as well as neurons (114). VIP receptors have two subtypes, the vasoactive intestinal peptide receptor type 1 (VPAC1) and type 2 (VPAC2), through which VIP transmitted the biological functions (115).

In the respiratory field, VIP has an anti-inflammatory and bronchodilator effect. A previous study reported that plasma levels of VIP was lower in patients experiencing an asthma exacerbation than in healthy subjects (116). In contrast, VIP levels in plasma negatively correlated with peak expiratory flow (PEF) improvement, indicating its usefulness as a possible predictor of responsiveness to bronchodilator therapy (116). In a double blind, randomized, placebo controlled, crossover trial in patients with moderate asthma, inhaled VPAC2 agonists induced more rapidly an improvement in FEV1 than placebo, but not as rapidly as inhaled formoterol. Additionally, the sustained bronchodilator effect of the VPAC2 agonist was shorter than that of formoterol, while there were no differences in adverse events between both groups. Thus, the superiority of this VPAC2 agonist over formoterol could not be supported in this study (117). Since this report, no reports about the effect of VIP agonists on asthmatics have been published.

Regarding anti-inflammatory effects, treatment with a VIP derivative suppressed the infiltration of inflammatory cells in the lungs and reduced epithelial wall thickness, as well as a significant reduction of macrophages and eosinophils in BALF, in an OVA-challenged rat model (118). However, a later study demonstrated that mice treated with VPAC2 antagonist following OVA challenge elicited a reduction in BALF CD4^+^ T cell numbers, IL-5 mRNA expression in CD4^+^ T cells and IL-13 production from lung ILC2 without altering total lung ILC2 numbers. In contrast, treatment with VPAC1 antagonist did not affect these parameters (100). VPAC1 and VPAC2 are both expressed on lung and intestinal ILC2, identified by qPCR. In an enteron, its neuron-derived VIP acts on VPAC1-expresing epithelial stem cells to suppress both their proliferation and differentiation of secretory system. Additionally, inhibition of this pathway enhanced production of IL-25 through the proliferation of tuft cells, subsequently leading to indirect activation of ILC2 and induction of T2 immune response (119). Thus, VPAC1 and VPAC2 may have opposing effects on induction of T2 inflammation through binding of VIP. Moreover, as for a functional assay of intestinal ILC2 *in vitro*, treatment with VIP or VPAC2 agonist further increased IL-5 production by stimulation with IL-7 (120). Also, the transcript levels of IL-5 and IL-13 in Th2 cells were increased by VIP treatment (100). These results suggest that ILC2 and Th2-driven airway eosinophilic inflammation is enhanced by VIP signaling via VPAC2 rather than VPAC1. On the other hand, VIP levels in BALF were elevated in OVA-challenged mice, whereas silencing airway sensory neurons by QX-314 reduced levels of VIP, as did CGRP (100, 116). Interestingly, IL-5 is known to be a potent inducer of eosinophilia, and it was also found that cultured nodose ganglion neurons expressed receptors for IL-5R, and IL-5 stimulation provoked VIP release from nodose ganglion neurons *in vitro* (100). Therefore, Th2 cell- and ILC2-derived IL-5 may contribute to VIP release from sensory neurons, potentially forming a positive feedback loop in the lungs (**Figure 2C**). These murine findings indicate that sensory neuron-derived VIP plays a crucial role in the induction of airway eosinophilic inflammation, while human evidence remains limited. On the other hand, early studies including the clinical trial demonstrated that VIP had inhibitory effects on airway inflammation and lung function. These discrepancies may reflect differences in receptor distribution and immune cell composition. Accordingly, current evidence supports a dual and highly context-dependent role for VIP in inflammatory regulation. However, these findings from murine ILC2 do not constitute direct evidence linking VIP signaling to the activation of human ILC2s. Therefore, the translational relevance of VIP-mediated neuroimmune interactions on ILC2 function requires further validation in human studies.

## 5 Conclusions

ILC2 express receptors for a variety of factors, such as lipid mediators, neurotransmitters, neuropeptides, and sex hormones. These factors modulate ILC2 function as well as epithelial-derived cytokines. In this review, we focused on the impact of neuropeptides and neurotransmitters on ILC2 in asthma pathophysiology (**Figure 4**; **Table 1**). In general, neuropeptides and neurotransmitters are released from central and peripheral nerve systems, but these molecules are mainly derived from peripheral local nerve systems that act on the airways to induce asthma symptoms. Our data from Ju and Nagashima et al. shows that in patients with mild asthma, airway eosinophilia in response to inhaled allergen challenge is positively correlated with airway levels of NMUR1^+^ ILC2 as early as 7 h post allergen. Our *in vitro* data also showed that NMU can induce the production of IL-5 and IL-13 from ILC2 within 6 h of stimulation compared to IL-33, which was only effective over 24 h of co-culture, although the effect of NMU on ILC2 activation was smaller than that of IL-33 (48). This suggests that neuropeptides such as NMU may contribute to the early initiation of ILC2 in asthma. Moreover, as mentioned in respective sections above, other neuropeptides and neurotransmitters can be shown to function as an amplifier or suppressor of T2 inflammation directly or indirectly *in vitro* and *in vivo*. Thus, neural signals play a key role in inducing ILC2s independently or synergistically, thereby contributing to the development of asthma. On the other hand, it has been reported that ILC2-derived ACh and CGRP may act on ILC2 themselves, and that ILC2-derived IL-5 can act on neurons in studies in mouse and human (92, 100, 113). These actions may provide a positive feedback loop involving ILC2 itself or between ILC2 and neurons, thereby contributing to the persistence of airway inflammation. However, there is insufficient evidence that ILC2 is involved in a feedback loop during chronic airway inflammation. Therefore, this view requires further examination. Currently, most evidence regarding the impacts of neuropeptides and neurotransmitters on asthma pathogenesis is obtained from mouse studies. Recently, some human data has been accumulating, and in clinical practice, drugs targeting CGRP, dopamine, and serotonin have already been used for a variety of neurological and psychiatric disorders. In contrast, as for therapeutic agents for asthma, short-acting muscarinic antagonists (SAMA) and LAMA, short-acting β_2_ agonist (SABA) and LABA. SAMA and LAMA work as a bronchodilator and inhibitor of mucus production, while SABA and LABA act as bronchodilators, the effects on airway inflammation remains unclear in human systems. Recently, we showed that, in line with the mouse studies, NMU functioned as a rapid activator for airway ILC2 via NMUR1 in mild asthmatics (48). In addition to NMU, other neuropeptides and neurotransmitters have been reported to act directly or indirectly on ILC2 in murine studies such as CGRP and VIP. However, direct evidence demonstrating their contributions to airway inflammation in human asthma remains limited for many neurotransmitters and neuropeptides. Therefore, further translational investigations are required to determine the extent to which these factors are involved in ILC2-mediated airway inflammation in asthma patients. As the understanding of the effects of neuropeptides and neurotransmitters on airway ILC2 in asthmatics increases, there is a potential to unveil novel therapeutic targets for this disease in the future.

**Figure 4:**
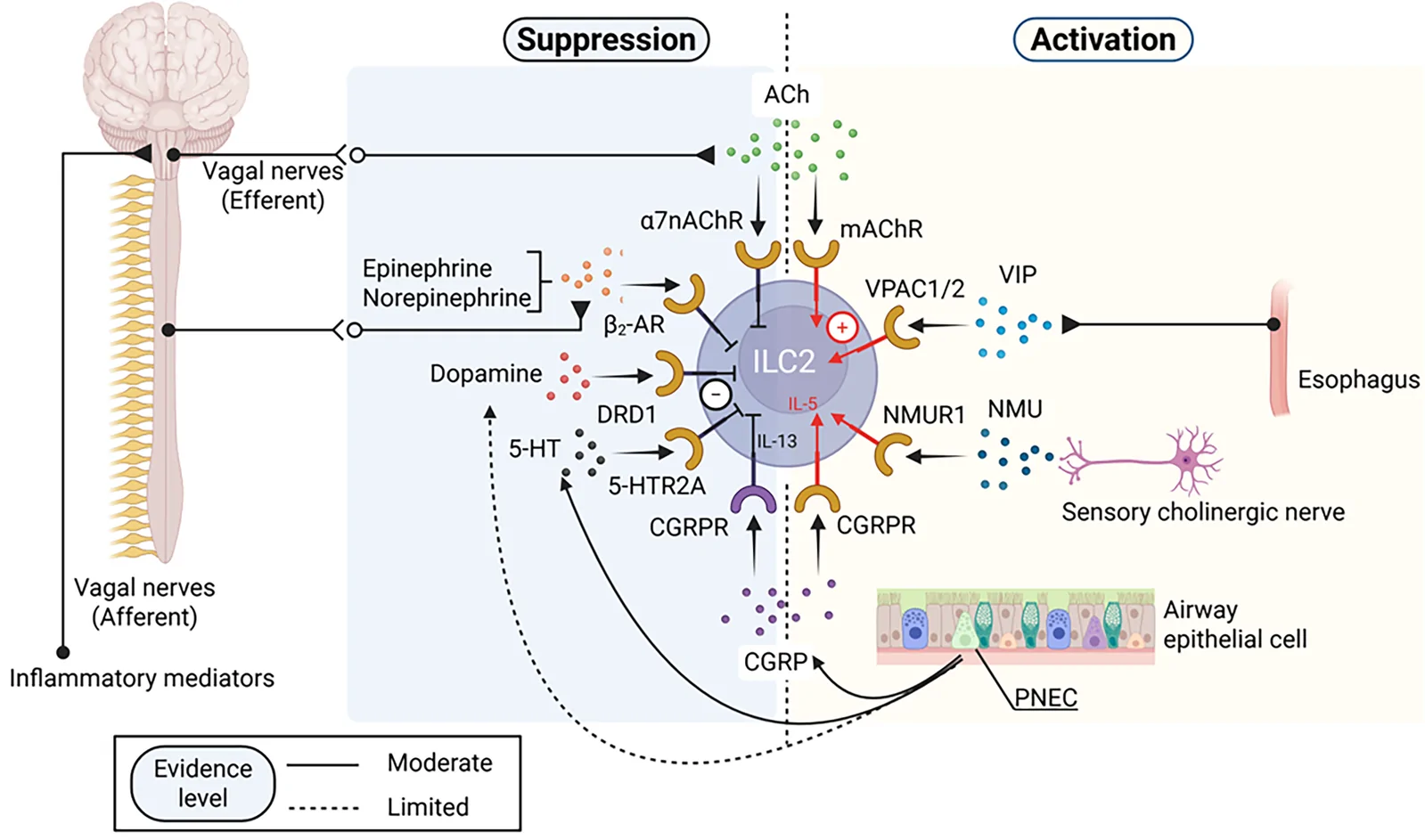
Summary of ILC2-expressing receptors for neuropeptides and neurotransmitters. ILC2 are activated or suppressed by neuropeptides and neurotransmitters through respective receptors. ACh, acetylcholine; α7nAChR, α7 nicotinic acetylcholine receptor; CGRP, calcitonin-gene related peptide; β2-AR; β2 adrenergic receptor; CGRPR, calcitonin-gene related peptide receptor; DRD1, Dopamine receptor D1; ILC2, group 2 innate lymphoid cell; 5-HT, 5-hydroxytryptamine; 5-HTR2A, 5-HT2A receptor; mAChR, muscarinic acetylcholine receptor; NMU, Neuromedin-U; NMUR1, Neuromedin-U receptor 1; PNEC, pulmonary neuroendocrine cells; TSLP, thymic stromal lymphopoietin; VIP, vasoactive intestinal peptide; VPAC1/2, vasoactive intestinal peptide receptor type 1/2.

**Table 1:** Overview of neuropeptides and neurotransmitters regulating ILC2s.

Mediator	Major source	Receptor on ILC2	Direct/ indirect effect on ILC2	Activating/ inhibitory	Experimental evidence on ILC2	Potential therapeutic relevance	Limitations
NMU (35, 39–43, 47–49)	•Sensory neurons •Cholinergic neurons	NMUR1	Direct	↑	Mouse *in vivo* Mouse *in vitro* Human *ex vitro*	NMUR1 agonists (preclinical)	•Limited human evidence
Norepinephrine (56)	•Sympathetic neurons	β_2_ adrenergic receptor	Direct	↓	Mouse *in vivo*	SABA LABA	•Limited human evidence
Dopamine (61)	•Central nerves •Peripheral tissues •PNEC	DRD1	Direct	↓	Mouse *in vivo* Mouse *in vitro* Human data	No established ILC2-targeted therapeutic strategy	•Limited human evidence
Serotonin (69, 71)	•Enterochromaffin cells •PNEC	5-HTR2A	Direct	↓	Mouse *in vivo* Mouse *in vitro* Human data	No established ILC2-targeted therapeutic strategy	•Contradictory findings among sources •Limited human evidence
Ach (82, 83, 87–92)	•Cholinergic neurons •ILC2	mAChR	Direct/ Indirect	↑	Mouse *in vivo* Mouse *in vitro*	LAMA	•Limited human evidence
	•Cholinergic neurons	α7nAChR	Direct	↓		α7nAChR agonists (preclinical)	
CGRP (102, 106, 107, 110–113)	•Sensory neurons •PNEC •ILC2	CALCRL/RAMP1	Direct	Biphasic	Mouse *in vivo* Mouse *in vitro*	Anti-CGRP mAbs (not approved for asthma)	•Contradictory findings among studies •Limited human evidence
VIP (100, 119, 120)	•Sensory neurons	VIPR1/VIPR2	Direct/ Indirect^†^	Biphasic	Murine *in vitro*	No established ILC2-targeted therapeutic strategy	•Limited human evidence

ACh, Acetylcholine; α7nAChR, α7 nicotinic acetylcholine receptor, CGRP, Calcitonin-gene related peptide; DRD1, Dopamine receptor D1ILC2, Group 2 innate lymphoid cell; LABA, Long-acting β2 agonist; LAMA, Long-acting muscarinic receptor antagonist; mAb, monoclonal antibody; mAChR, muscarine 4–5 acetylcholine receptor; NMU; Neuromedin-U; PNEC, Pulmonary neuroendocrine cell; SABA, Short-acting β2 agonist; VIP, vasoactive intestinal peptide; 5-HTR2A, 5-HT2A receptor; ^†^Indirect effects have been reported only in the intestine. ↑, Activation; ↓, Inhibition
